# Data partitioning and correction for ascertainment bias reduce the uncertainty of placental mammal divergence times inferred from the morphological clock

**DOI:** 10.1002/ece3.4921

**Published:** 2019-01-30

**Authors:** Ian V. Caldas, Carlos G. Schrago

**Affiliations:** ^1^ Department of Genetics Federal University of Rio de Janeiro Rio de Janeiro Brazil

**Keywords:** ascertainment bias, character coding, evolutionary radiation, morphological clock, morphology model

## Abstract

Bayesian estimates of divergence times based on the molecular clock yield uncertainty of parameter estimates measured by the width of posterior distributions of node ages. For the relaxed molecular clock, previous works have reported that some of the uncertainty inherent to the variation of rates among lineages may be reduced by partitioning data. Here we test this effect for the purely morphological clock, using placental mammals as a case study. We applied the uncorrelated lognormal relaxed clock to morphological data of 40 extant mammalian taxa and 4,533 characters, taken from the largest published matrix of discrete phenotypic characters. The morphologically derived timescale was compared to divergence times inferred from molecular and combined data. We show that partitioning data into anatomical units significantly reduced the uncertainty of divergence time estimates for morphological data. For the first time, we demonstrate that ascertainment bias has an impact on the precision of morphological clock estimates. While analyses including molecular data suggested most divergences between placental orders occurred near the K‐Pg boundary, the partitioned morphological clock recovered older interordinal splits and some younger intraordinal ones, including significantly later dates for the radiation of bats and rodents, which accord to the short‐fuse hypothesis.

## 1 INTRODUCTION

The radiation of placental mammals culminated in a variety of body forms and life histories (Feldhamer, Drickamer, Vessey, Merritt, & Krajewski, 2007). This process has been traditionally linked to the Cretaceous–Paleogene (K‐Pg) mass extinction, with the disappearance of nonavian dinosaurs and the subsequent vacancy of ecological niches (Carroll, 1997). Archibald and Deutschmann (Archibald & Deutschman, 2001) proposed three distinct hypotheses of the tempo and mode of placental evolution in relation to the K‐Pg event. Firstly, the explosive hypothesis postulates that Placentalia arose from stem eutherians in the early Paleogene and rapidly underwent explosive radiation; therefore, no placentals existed in the Cretaceous according to this model. Alternatively, the long‐fuse hypothesis postulates the origin of Placentalia before the K‐Pg event, with interordinal radiation and extinct stem lineages deep in the Cretaceous, but with intraordinal diversification in the early Paleogene. Finally, the short‐fuse hypothesis also argues in favor of a placental origin in the Cretaceous but postulates a small role for the K‐Pg event: some orders underwent intraordinal radiation in the Cretaceous, while others in the Paleogene.

There is a long‐standing debate on the timing of the crown placental radiation (Beck & Lee, 2014; Bininda‐Emonds et al., 2007; dos Reis et al., 2012; O'Leary et al., 2013; Puttick, Thomas, & Benton, 2016). In particular, molecular and paleontological sources of evidence provide conflicting results on the evolution of early placental mammals. Paleontological evidence supports either the explosive or the long‐fuse hypothesis, depending on the phylogenetic assignment of fossils of early placental mammals (Wible, Rougier, Novacek, & Asher, 2007). On the other hand, molecular dating studies recovered older dates when compared to the expectation from the fossil record, with early analysis supporting the short fuse (Kumar & Hedges, 1998), while later studies have corroborated the long‐fuse hypothesis (dos Reis et al., 2012; Meredith et al., 2011), but never the explosive model. Bayesian methods allow for a direct quantitative comparison between both types of data, morphology, and molecules, by using probabilistic models of morphological evolution, such as the Mk model (Lewis, 2001).

The Mk models the evolution of discrete‐state morphological characters as a stochastic process, namely, a Markov chain with identical equilibrium frequencies of states and equal instantaneous transition probabilities. The Mk is thus a general, data‐independent version of the classic Jukes–Cantor model of nucleotide evolution (Jukes & Cantor, 1969). Adoption of the Mk model in probabilistic analysis of morphology remains controversial, as doubts were cast on whether such a simple framework can accurately account for the intricacies of phenotypic evolution (Brown, Parins‐Fukuchi, Stull, Vargas, & Smith, 2017; Dávalos, Velazco, Warsi, Smits, & Simmons, 2014; Puttick, O'Reilly, Oakley et al., 2017; Schrago, Aguiar, & Mello, 2018). A fundamental criticism rests on the assumption that morphological characters evolve under a common mechanism, that is, the rate of evolution at each branch is linearly correlated among characters (Goloboff, Pittman, Pol, & Xu, 2018; Tuffley & Steel, 1997); therefore, if a given character evolves faster in some lineage, it is expected that another unrelated character will also evolve relatively faster in this same lineage. This feature may not be the norm of phenotypic evolution (Lee, 2016). Moreover, acquisition of discrete morphological data tends to favor those characters that vary among the terminals analyzed, yielding a biased sampling of data known as ascertainment bias (Lewis, 2001). Nonetheless, the Mk model uniquely permits several advanced statistical molecular phylogenetics analyses to be carried out with traditional morphological data.

Standing out among such approaches is the morphological clock. Applying relaxed clock methods to phenotypic data was initially conducted under a total‐evidence context, consisting of a combined analysis of morphological data as well as molecular data (Pyron, 2011; Ronquist, Klopfstein et al., 2012a). In this sense, fossil taxa were incorporated as tree tips with fixed ages. Subsequent attempts have been made at a pure morphological clock analysis, enabling the estimation of divergence times using phenotypic data alone (Lee, Cau, Naish, & Dyke, 2014; Schrago, Mello, & Soares, 2013). Under Lewis model, dating using morphological data is carried out with the same analytical approaches employed to molecular matrices, such as the relaxed clock. Therefore, node ages are estimated under a Bayesian framework without relying on the constancy of evolutionary rates along branches (Bromham et al., 2018). As with molecules, rate priors are used to model the evolution of rates along branches assuming either independent (Drummond, Ho, Phillips, & Rambaut, 2006) or correlated processes (Kishino, Thorne, & Bruno, 2001; Thorne, Kishino, & Painter, 1998).

Considerable effort has been spent on understanding the factors that influence posterior distributions of Bayesian divergence time estimates (Dos Reis & Yang, 2013). For the relaxed molecular clock, the larger the variation in rates across lineages, the larger is the uncertainty of inferred divergence times. This outcome may be circumvented by increasing the number of partitions the data is divided in, as each partition is treated as an independent loci (dos Reis et al., 2012; Rannala & Yang, 2007; Zhu, Reis, & Yang, 2015) In this case, the rate of decrease of the variance of age estimates is roughly the reciprocal of the number of partitions (Zhu et al., 2015). Whether this property also holds for phenotype matrices is still unexplored. Morphological data are intrinsically different from biological sequences, and recent works have demonstrated that the statistical behavior of phylogenetic methods using molecular datasets cannot be readily replicated with phenotypic data (O'Reilly, Puttick, Pisani, & Donoghue, 2017; Puttick, O'Reilly, Tanner et al., 2017; Puttick et al., 2016; Schrago et al., 2018). We verify here whether data partitioning affects uncertainty of age estimates in the case of the pure morphological clock. This was carried out employing an experimental design that allowed for comparisons among discrete phenotypic, molecular, or both sources of data. We do so in the context of placental mammals, in the hope of uncovering novel information about their much‐disputed radiation.

## 2 MATERIALS AND METHODS

Phenotypic data were taken from the matrix of O'Leary et al. (2013), which was composed of 4,541 discrete‐state scored morphological characters. Molecular data were obtained from the alignment of Meredith et al. (2011). It consisted of a supermatrix of 26 gene fragments totaling 35,603 base pairs, 2,573 of which comprised untranslated regions. In all analyses, this molecular matrix was divided into 12 partitions according to the best scheme suggested by the PartitionFinder software (Lanfear, Calcott, Ho, & Guindon, 2012). For the sake of comparison between results from molecules and morphology, we composed a dataset consisting of 40 extant mammal species, for which both molecular and morphological data were available. Thus, extinct taxa were not included in our matrix. All ambiguous observations, characters coded as a combination of two or more states, were treated as unknown states. In total, seven independent divergence time analyses were run using different data and analytical procedures, namely, (a) molecular data alone; (b) unpartitioned morphological data with and (c) without ascertainment bias; (d) partitioned morphological data with and (e) without ascertainment bias; and (f) combined molecular and morphological data with and (g) without ascertainment bias.

Tree topology was inferred combining both molecules and morphology in MrBayes 3.2.2 (Ronquist, Teslenko et al., 2012b). Each molecular partition was allowed to evolve independently through the GTR model, with gamma‐distributed rate variation across sites and five rate categories. Because of computational limitations, morphological characters with more than 10 states were removed from the phenotypic data, leaving a total of 4,533 characters. Morphological data were assumed to evolve under the Mk model (Lewis, 2001), with a 5‐category gamma‐distributed rate variation across sites. Default priors were used across all analyses. The Markov chain Monte Carlo (MCMC) algorithm was run for a variable number of generations and burn‐in periods, which were set to achieve a minimum of 8 million chains and effective sample sizes (ESS) > 200. Sampling of chains was performed every 2,500th cycle. A maximum clade credibility tree was built with the resulting posterior distribution of trees. The inferred Bayesian tree topology was fixed in all subsequent divergence time analyses, avoiding uncertainty from topological inference.

BEAST 1.10 (Suchard et al., 2018) was used to estimate divergence times between species using both morphology and molecular data. For morphology, besides analyzing the unpartitioned morphological matrix, we also estimated divergence times using 4 partitions according to the major anatomical divisions proposed by O'Leary et al. (2013), which consisted of 1,284 cranial, 1,451 dental, 925 postcranial, and 881 soft‐tissue characters.

In both sources of data, partitions were allowed to have their own substitution models and clock rates. The GTR model with 4 gamma categories was used for molecular data, while the Mk model with 4 gamma categories was used for the morphological data. The uncorrelated lognormal clock (Drummond et al., 2006) was assumed for all partitions. The birth–death process with species sampling (Stadler, 2009) was used as the prior on uncalibrated divergence times. For substitution model parameters, default prior distributions were used.

The Mk model was implemented in the XML format for BEAST (Pyron, 2011; Schrago et al., 2013). The format requires the user to specify how many states are permitted for each character. Two different schemes were used to specify the number of possible character states: in the first scheme, the number of states was defined by how many states the character showed in the reduced 40‐taxa matrix. In the second scheme, henceforth referred to as the reduced ascertainment bias, the number of states was taken directly from the character notes of O'Leary et al. (2013). This scheme allowed characters that are invariable in the sample of 40 taxa to be included in the analysis and considered multi‐state, acting as invariable sites in traditional DNA phylogenetics and lessening the effects of ascertainment bias (Lewis, 2001). All MCMC chains were run for 100 million generations, being sampled every 10,000th cycle. Analyses were run in the CIPRES Science Gateway (Miller, Pfeiffer, & Schwartz, 2010). Posterior distributions of divergence times were calculated using the Tree Annotator software (Drummond, Suchard, Xie, & Rambaut, 2012), removing 20% of the trees as burn‐in.

We used a total of 9 fossil calibrations (Table 1) following the recommendations from the recent survey of Benton et al. (2015). Calibration uncertainty was accommodated in all cases by a normal distribution, with 95% probability density delimited by the suggested minimum and maximum age of the relevant taxon split by the authors. Although calibration assignment and shape are known to impact age estimates (Sauquet et al., 2012), our analysis focused on comparing the relative performances of datasets under different partitioning schemes but maintaining the same set of calibrations for all analyses. Therefore, we note that eventual doubts on fossil assignments, or future reinterpretations of their phylogenetic position, will not affect our conclusions on the relative performance of partitioning schemes, as the same set of calibration priors were applied in all comparisons carried out.

**Table 1:** Calibration information used in this study

Divergence	Mean and standard deviation of normal prior
Caniformia/Feliformia	*μ* = 51.65, σ = 7.3
Rodentia/Lagomorpha	*μ* = 110.3, *σ* = 27.8
Rodentia/Primates	*μ* = 112.95, *σ* = 26.4
Euarchontoglires/Laurasiatheria	*μ* = 112.95, *σ* = 26.4
Xenarthra/Boreoeutheria	*μ* = 112.95, *σ* = 26.4
Afrotheria/Boreoeutheria	*μ* = 112.95, *σ* = 26.4
Australiadelphia/Ameridelphia	*μ* = 89.45, *σ* = 21.4
marsupials/placentals	*μ* = 162.95, *σ* = 3.4
Crown Mammalia	*μ* = 183.2, *σ* = 9.8

Note

All calibrations were assigned as normal priors with 95% highest probability interval set to be bounded by the maximum and minimum limits suggested by Benton et al. (2015).

The infinite‐sites plot was used to investigate the impact of sequence and calibration information in reducing amount of uncertainty (widths of posterior credibility intervals) in divergence time estimates (Rannala & Yang, 2007). As sequence length goes to infinity, the mean of the posterior distribution of divergence times and credibility interval widths are expected to converge to a linear relationship. They were plotted against each other and a line through the origin was fitted to the data using the R statistics package.

## 3 RESULTS

The inferred tree topology from combined data recovered all ordinal and supraordinal clades of Placentalia as monophyletic (Supporting information Figure S1). Euarchontoglires was recovered as sister to Laurasiatheria, forming the Boreoeutheria, which is sister to Xenarthra. The (Boreoeutheria + Xenarthra) clade was sister to Afrotheria.

**Figure 1:**
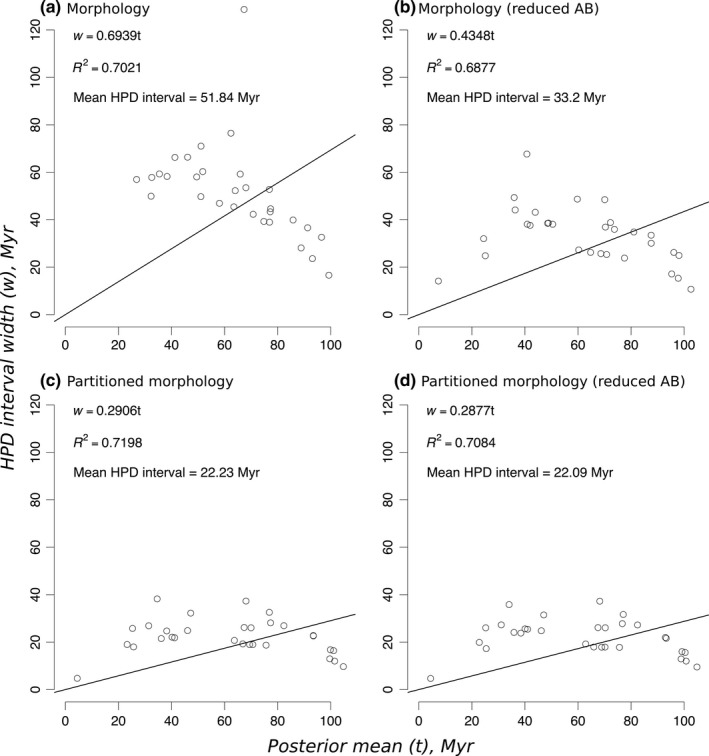
Infinite‐sites plot of morphological analyses. AB: ascertainment bias; HPD: highest posterior density; Myr: million years

Morphological clock estimates were influenced by the partitioning scheme and data treatment (Figure 1; Supporting information Figures S1–S6). Partitioning data resulted in 57% reduction of HPD interval widths. Accounting for ascertainment bias also had a substantial impact on posterior uncertainty of divergence time estimates. Compared to untreated data, reducing ascertainment bias yielded an average 36% reduction of HPD interval widths. Both partitioning and reducing ascertainment bias produced the same effect as partitioning alone, bringing a 57% reduction in uncertainty as well (Figure 1). All regressions of node ages with associated uncertainties inferred from morphological data alone have comparable linear fits (*R* ^2^ ~ 0.7).

**Figure 2:**
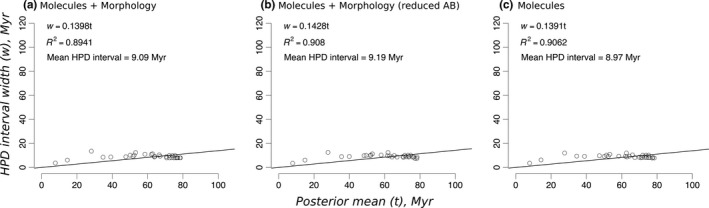
Infinite‐sites plot of combined and molecular analyses. AB: ascertainment bias; HPD: highest posterior density; Myr: million years

Divergence time estimates obtained from molecular data alone presented an average HPD interval width of ~9 Ma, which was the same value retrieved using the combined analysis of morphological and molecular data (Figure 2). The linear relationship between time estimates and HPD widths also indicated that molecular and combined analyses performed similarly, both yielding HPD interval widths that were roughly 14% of the estimated divergence time.

**Figure 3:**
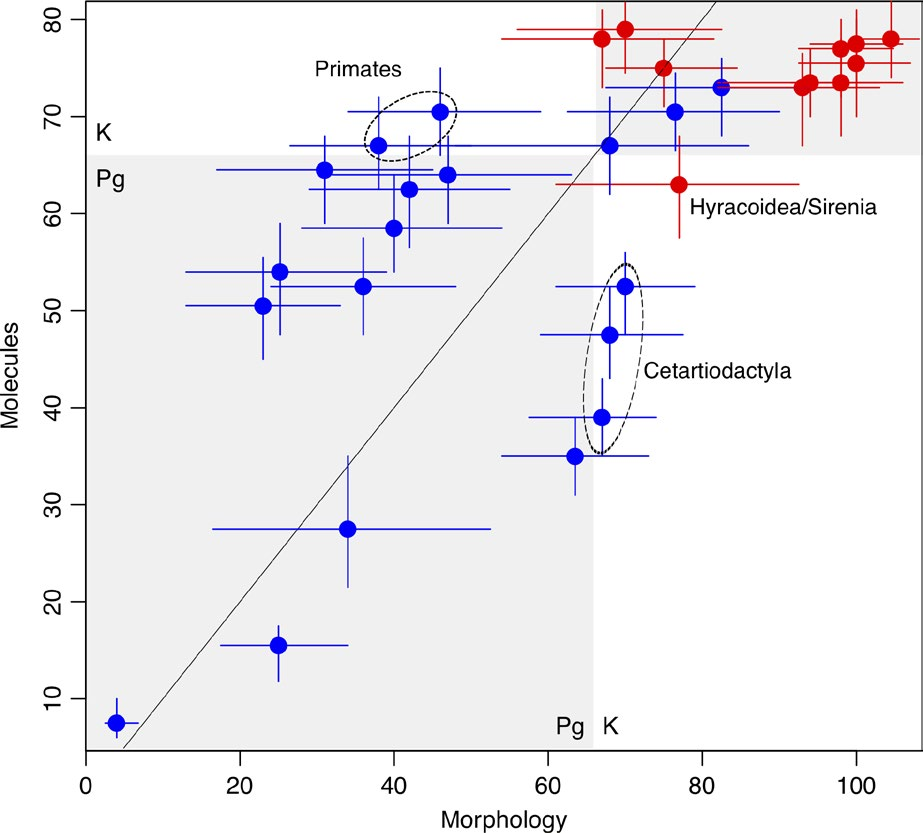
Comparison between divergence times estimated from the morphological and molecular clocks. Blue and red solid circles represent within and between‐order divergences, respectively. Bars indicate the 95% HPD intervals. The shaded area encompasses the values in which both data sources yielded divergence time estimates lying in the same geological period. Points outside this area depicts the age estimates in which molecules and morphology were in conflict regarding the geological period that they took place (e.g., within‐order splits of Primates and Cetartiodactyla)

Using the morphological clock, most interordinal divergence times inferred from morphology were older, whereas intraordinal splits were dated younger, although both sources of data placed the ages of those early interordinal splits in the Cretaceous period (Figure 3). The only exception was the Hyracoidea/Sirenia divergence within Afrotheria, in which a conflict was found between molecules and morphology, as the molecular clock placed the split in the Paleogene. Compared to analyses including molecular data, morphology also recovered older divergence times for Cetartiodactyla intraordinal divergences, with credibility intervals extending to the K‐Pg period boundary. On the other hand, molecular data placed primate intraordinal divergences in the Cretaceous, while morphology placed it in the Paleogene (Figure 3).

## 4 DISCUSSION

We have shown that partitioning of morphological data reduced the uncertainty of posterior divergence time estimates using the relaxed morphological clock, a behavior that has been previously reported with the molecular clock (Zhu et al., 2015). We employed a very large phenotypic matrix, divided into four partitions according to broad anatomical categories. Smaller phenotypic matrices are, however, more common in the literature, as character coding is a laborious process that requires specialized training (Scotland, Olmstead, & Bennett, 2003). Depending on partition length, increasing the number of partitions in molecular matrices should be more efficient in reducing uncertainty than increasing total amount of data (Zhu et al., 2015). It is worth investigating whether careful partitioning can overcome the limited size of such matrices and produce more accurate divergence time estimates employing the morphological clock.

Zhu et al. (2015) listed three major factors contributing to the uncertainty of divergence time estimates from molecular data: sampling error due to short sequences, evolutionary rate variation due to the relaxed clock, and inherent uncertainty of fossil calibrations. Here, we propose a fourth component: character scoring, which ultimately generates ascertainment bias. Phenotypic characters, lacking a common code or alphabet, are scored by human specialists and are vulnerable to subjectivity (Scotland, Pennington, & Association, 2000). We have also shown that reducing ascertainment bias had a significant impact in reducing posterior uncertainty of divergence times. The effect of ascertainment bias, and character coding in general, has been largely unexplored when comparing the relative performances of phylogenetic methods using probabilistic models of morphology (Brown et al., 2017; O'Reilly et al., 2016; Schrago et al., 2018). Because morphological matrices frequently consist only of those characters that vary among terminals, the rate of morphological change, which ultimately impacts divergence time estimation, may be biased. Therefore, correcting for ascertainment bias likely reduces the uncertainty of age estimates by decreasing rate variation among branches. Previous analyses have also reported that divergence time estimates of the age of crown Placentalia using the morphological clock were older than those inferred from molecular data (Beck & Lee, 2014; Puttick et al., 2016). This study is the first morphological analysis to recover younger dates for several mammalian divergences. It is generally accepted that phenotypic changes diagnostic of lineages will accumulate after their complete genetic isolation (Nei & Kumar, 2000), while genetic changes may start well before than the speciation time (Capellão, Costa‐Paiva, & Schrago, 2018; Edwards & Beerli, 2000). Therefore, the relative pace of the morphological clock should be slower than the molecular clock. Under the clock, mean rate differences alone do not explain the discrepancies found between molecules and morphology to estimate the mammalian timescale, as they were scaled using the same set of calibration priors. Incompatibilities between morphology and molecules indicate that modeling morphological changes bears difficulties that are not mirrored in evolutionary models of biological sequences (Rineau, Grand, Zaragüeta, & Laurin, 2015; Schrago et al., 2018). It is worth mentioning that, although differences were found between age estimates from morphology and molecules, on average, both timescales were similar. The mean ages of interordinal divergences were 74.6 and 74.8 Ma for molecules and morphology, respectively, while the mean ages of intraordinal splits were 50.3 Ma (molecules) and 51.5 Ma (morphology). To our knowledge, this is the first analysis to recover this temporal correspondence.

In this study, we analyzed solely extant taxa, for which data are more easily accessible. Further investigation is required to verify whether our results hold when fossils are used as terminal taxa. Even with the limitations of morphological matrix coding, we believe the morphological clock is a promising avenue of research in phylogenetics. We showed that, when probabilistic models are used, the statistical behavior of morphological data in divergence time inference is similar to that of molecular matrices. Future effort should focus on the treatment of phenotype‐specific attributes. For instance, it is unclear whether the diagnostic traits coded by morphologists suffer from the processes that generate gene tree/species tree discordance (Maddison, 1997), impacting divergence time estimates, or whether ascertainment bias is capable of fully explaining the alternative chronological scenario of mammal evolution provided by the morphological clock in previous analyses.

## CONFLICT OF INTEREST

None declared.

## AUTHORS CONTRIBUTION

All authors have designed the experiments, collected and analyzed data, interpreted the results, and wrote the paper.

## Data Availability

All data used in this study is publicly available in the Dryad repository.
