# Supplementary material for: Data partitioning and correction for ascertainment bias reduce the uncertainty of placental mammal divergence times inferred from the morphological clock
Source: Ecol Evol. 2019 Jan 30;9(4):2255–62. doi: 10.1002/ece3.4921 (PMC6392387; doi:10.1002/ece3.4921)

Supplementary figures of

**Data partitioning and correction for ascertainment bias reduce the uncertainty of placental mammal divergence times inferred from the morphological clock**

**Figure S1.** Phylogenetic tree estimated by MrBayes 3.2.2, with branch lengths measured in expected substitutions per site. Branch length is measured in million years.

**Figure S2.** Divergence time estimations for unpartitioned morphological data. Red nodes received calibrations. Branch length is measured in million years.


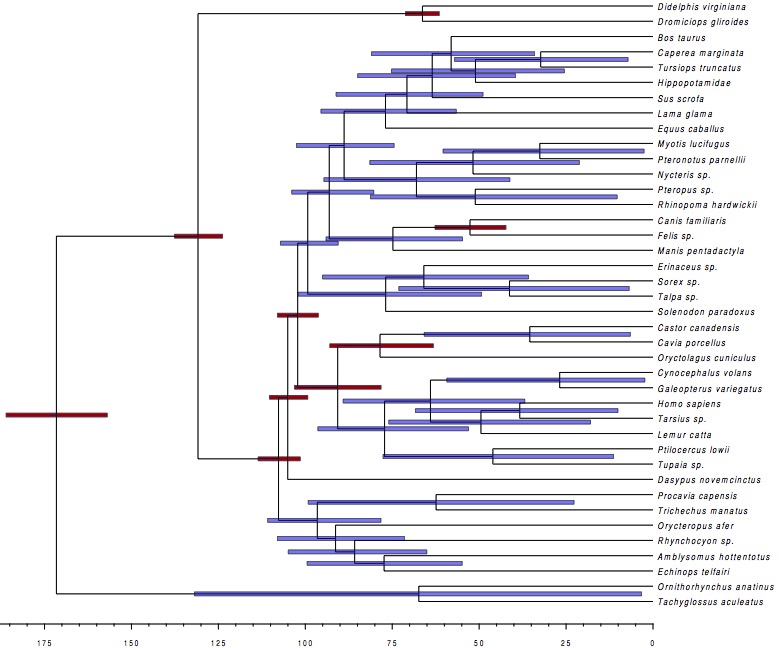


**Figure S3.** Divergence time estimations for morphological data, divided in 4 partitions. Red nodes received calibrations. Branch length is measured in million years.


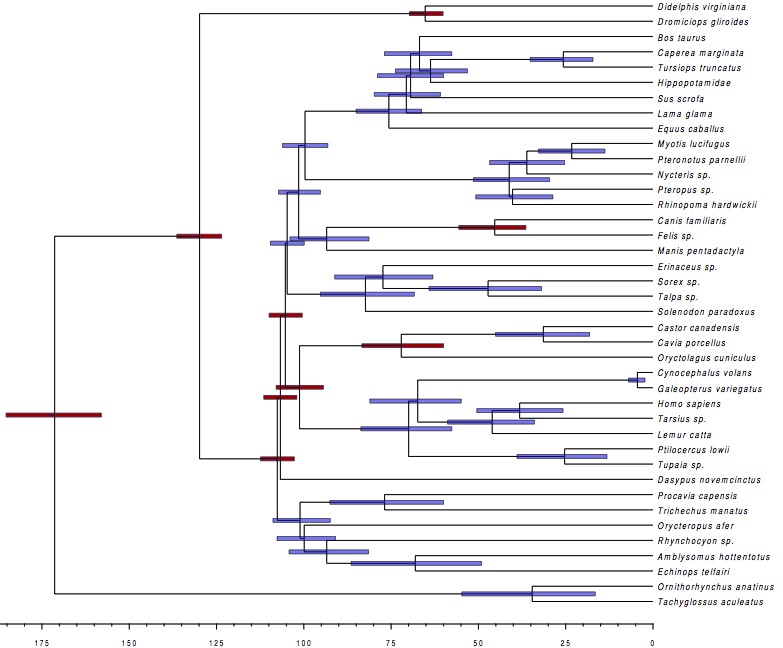


**Figure S4.** Divergence time estimations for unpartitioned morphological data, with reduced ascertainment bias. Red nodes received calibrations. Branch length is measured in million years.


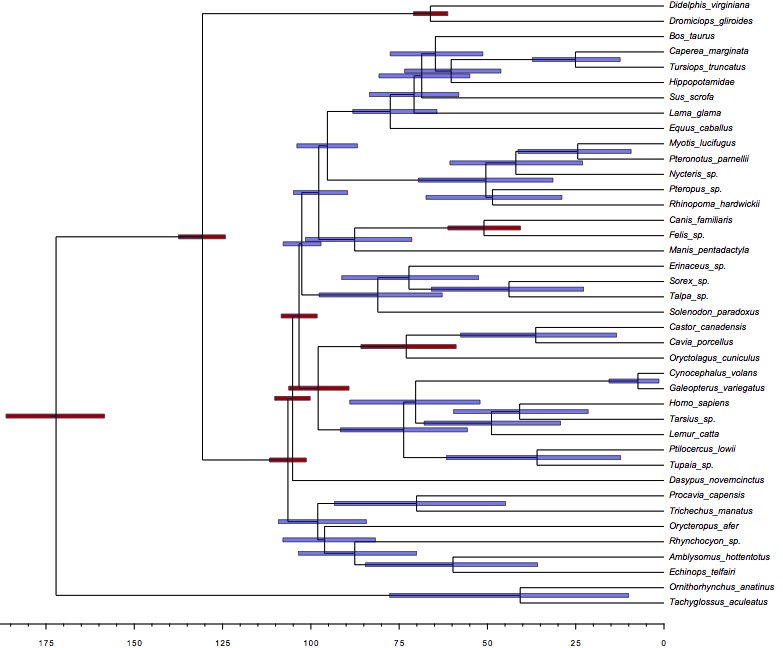


**Figure S5.** Divergence time estimations for morphological data, divided in 4 partitions, with reduced ascertainment bias. Red nodes received calibrations. Branch length is measured in million years.


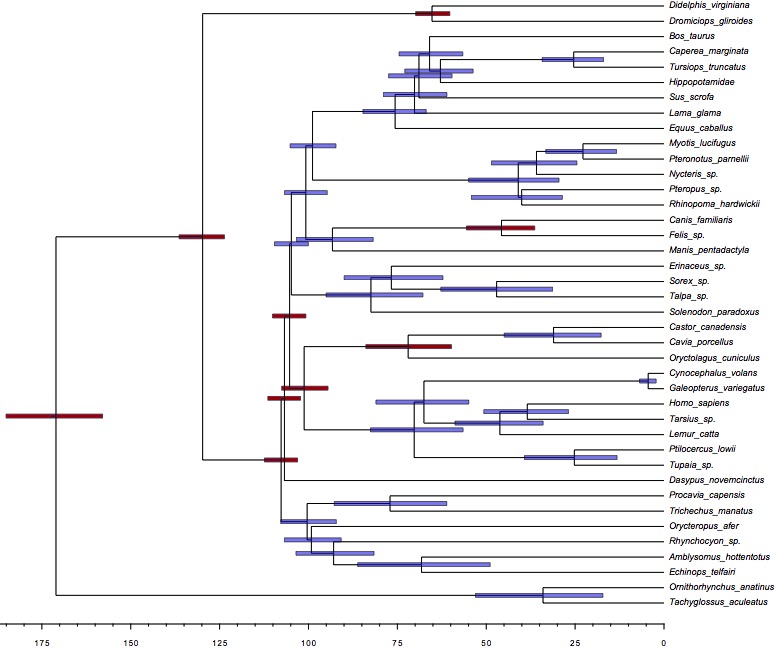


**Figure S6.** Divergence time estimations for combined molecular and (unpartitioned) morphological data. Red nodes received calibrations. Branch length is measured in million years.


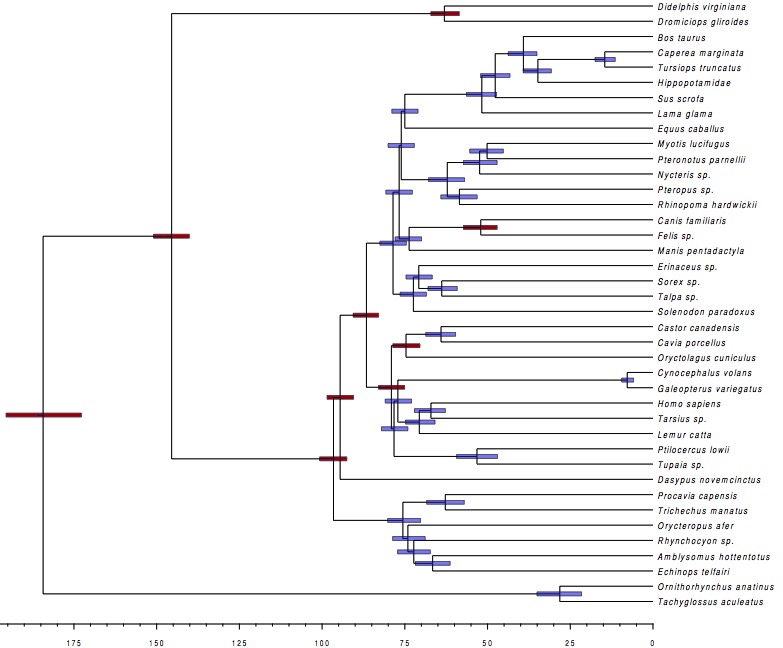

Supplement: Supplementary file 1 [file ECE3-9-2255-s001.docx]
